# Process Evaluation of a Regional Public Health Model to Reduce Chronic Disease Through Policy and Systems Changes, Washington State, 2010–2014

**DOI:** 10.5888/pcd12.140446

**Published:** 2015-03-19

**Authors:** Lina P. Walkinshaw, Caitlin Mason, Claire L. Allen, Thuy Vu, Paj Nandi, Patti Migliore Santiago, Peggy A. Hannon

**Affiliations:** Author Affiliations: Lina P. Walkinshaw, Claire L. Allen, Thuy Vu, Peggy A. Hannon, University of Washington, Seattle, Washington; Paj Nandi, Patti Migliore Santiago, Washington State Department of Health, Olympia, Washington.

## Abstract

**Introduction:**

Although the regionalization of public health systems has been well documented in the case of emergency preparedness, there is little literature on the application of regional approaches to other aspects of public health. From 2011 through 2014 the Washington State Department of Health implemented a Community Transformation Grant to support community-level policy and systems changes to decrease chronic disease risk factors and increase access to clinical preventive services. The Department of Health implemented the grant through a regional model, grouping 32 of the state’s 35 local health jurisdictions into 5 regions. Our process evaluation identifies the challenges and facilitators to Community Transformation Grant planning and implementation.

**Methods:**

We conducted 34 key informant interviews with people directly involved in the implementation of the Community Transformation Grant. We interviewed state and local partners, including representatives from each region, the Department of Health, external consultants, and regional partners. We collected data from October 2013 through July 2014.

**Results:**

Challenges for planning, building, and implementing a regional model for chronic disease prevention included stakeholder buy-in, regional geography, and communication; facilitators included shared regional history and infrastructure, strong leadership, collaborative relationships, shared vision and goals, sufficient funding, and direct technical assistance and training.

**Conclusion:**

Lessons learned in Washington State provide a foundation for other states interested in using a regional approach to reduce chronic disease risk. Policy and systems changes require adequate time, funding, and staffing. States and funders should work closely with local leaders to address these challenges and facilitators.

## Introduction

### The regionalization of public health work

Local health jurisdictions (LHJs) across the United States began exploring the regionalization of public health work though intrastate governance systems in the 1970s (1). States can achieve regionalization through coordinated organizational and personal networking, service standardization through uniformity of tools and protocols, or centralization of fiscal and administrative resources (2). Many US regional public health models stem from coordinated efforts to increase emergency preparedness after the attacks on September 11, 2001 (1), when many states developed regional emergency preparedness models to increase statewide coordination and ensure efficient allocation of federal resources (3,4).

Public health experts believe regional work can support increased efficacy and efficiency because larger public health delivery systems generally maintain stronger performance measures (4). Providing greater funding to fewer jurisdictions can also be cost effective (2,5); resource-sharing can eliminate duplication and increase the ability to address challenges that cross jurisdictional boundaries (4).

The potential for cost savings through regional public health models has prompted increased use; some states have explored the benefits of also regionalizing systems for chronic disease management and prevention (1,6). Since 2010, increased efforts by the Centers for Disease Control and Prevention (CDC) and the Robert Wood Johnson Foundation to regionalize public health services has expanded the scope of regional efforts beyond emergency preparedness and shaped evolving terminology; the term “regionalization” is now often used to describe the merging of departments or regional infrastructure, and regional efforts to partner across jurisdictional boundaries is referred to as “cross-jurisdictional sharing” (7). Yet despite increased regional efforts, little published literature documents the challenges and facilitators to implementing regional models for policy and systems changes or chronic disease control.

### Policy and systems changes to support chronic disease prevention

Given the substantial effects of political, social, and environmental factors on individual and community health, local, state, and national leaders are beginning to focus on policy and systems changes to address population-level chronic disease risk (8). The 2002 Institute of Medicine report *The Future of the Public’s Health in the 21st Century* identified the adoption of “healthy” policies as a necessary strategy for governments and regional partners to improve population health (9).

The 2010 Patient Protection and Affordable Care Act provides increased opportunities for LHJs to collaborate with regional partners to improve social, economic, and environmental factors that affect community health (7,10). The 2011 Public Health Accreditation Board’s national accreditation standards also encourage collaborative regional partnerships to ensure LHJs are providing communities with the 10 essential public health services (11). A LHJ that is unable to provide all of the essential services can meet accreditation standards by demonstrating collaborative agreements with partner agencies who provide said services (11). The Institute of Medicine also identifies the development of collaborative, nontraditional systems-wide partnerships as an area for public health action to reduce chronic disease (9).

Despite the potential effect of policy and systems changes and the opportunities to support this work through regional efforts, policy and systems change work remains a relatively new public health strategy (8). Coalitions and networks to address health through social and environmental policy began forming in the mid-1990s. Tobacco control and use prevention strategies as well as recent national healthy communities funding initiatives are shifting traditional program-based jurisdictional public health work toward broader systems-level approaches (10). The CDC’s Community Transformation Grants (CTGs) provided an opportunity for recipients to tackle policy and systems changes (12).

### Overview of Washington State’s Community Transformation Grant

In 2011, CDC awarded the Washington State Department of Health (WA DOH) a CTG to improve community health through policy, environment, and systems changes. The CTG focused on “expanding efforts in tobacco-free living, active living and healthy eating, quality clinical and other preventive services, and healthy and safe physical environments” (12). Examples of work implemented in Washington State through the CTG include tobacco-free college campuses, complete streets ordinances (to direct the development of city and county streets to be functional for pedestrians, bicyclists, and people of all abilities), community health worker programs, and regional healthy community policy platforms.

Washington State has a decentralized public health system, with 39 counties divided into 35 distinct LHJs. A decentralized system affords LHJs substantial influence and control over the provision of local public health services; however, it requires a high level of administrative funding to support each department because of overhead costs and necessary programs and services.

Growing fiscal constraints and challenges in maintaining equitable public health services across the state prompted WA DOH to implement the CTG through a regional model. WA DOH’s long-term vision was to use this regional model to develop a comprehensive statewide chronic disease prevention system, eventually funneling non-CTG funding through regions as well.

WA DOH designated 5 regions and 11 priority counties in their CTG proposal to CDC (Figure); participating counties approved each region; 3 LHJs applied for and received CTG funding separately and were not funded through WA DOH. Priority counties were selected on the basis of total and rural population size, geographic location relative to other target counties, and the prevalence of risk factors targeted by the grant; region formation was not based on prior cross-jurisdictional collaboration. WA DOH selected 1 LHJ to serve as the fiscal and administrative lead for each region and provided funding to support a regional coordinator. To streamline communication, WA DOH assigned each regional coordinator an internal WA DOH staff consultant who was responsible for connecting the coordinator with resources, subject matter expertise, training, and technical assistance from an external consulting firm. WA DOH selected the external consulting firm on the basis of the firm’s expertise and ability to facilitate regional partnerships. WA DOH determined that all regional communication regarding CTG would flow between the regional coordinators and their WA DOH consultants.

**Figure:**
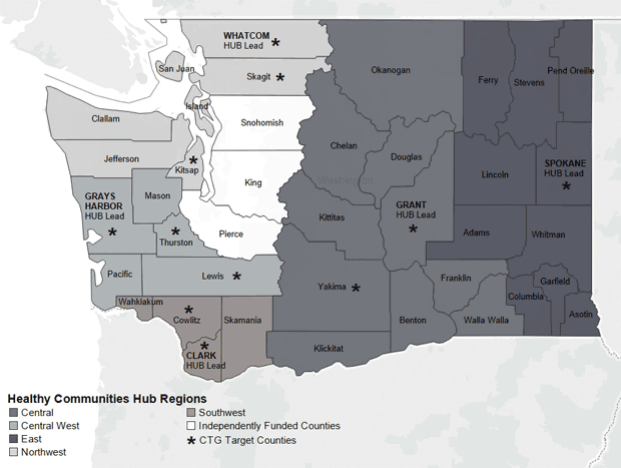
Regions used for Community Transformation Grant (CTG) implementation in Washington State, 2011–2014.

### Primary objectives of this evaluation

We conducted a process evaluation of CTG implementation in Washington to better understand the challenges and facilitators to implementing a statewide regional model to reduce chronic disease risk through policy and systems changes. WA DOH planned to use the evaluation results to make midcourse improvements. However, although CDC awarded the CTG as a 5-year grant, the federal government cut funding after year 3. Lessons learned from the implementation of the CTG in Washington State can support both Washington and other states in planning, building, and implementing regional models to reduce chronic disease through policy and systems changes. This evaluation also contributes to the literature on the implementation of regional public health work beyond emergency preparedness (4).

## Methods

From October 2013 to July 2014, we conducted a qualitative process evaluation of Washington State’s CTG implementation. Our data collection methods were document review (eg, grant application, meeting notes) and 34 semi-structured key informant interviews. The consolidated criteria for reporting qualitative research guided our methodology (13). The members of our evaluation team were not directly affiliated with CTG implementation.

### Interviews

We identified and interviewed 14 WA DOH leadership and staff members, 11 LHJ leadership and staff members, 4 external consultants, and 5 other regional partners, whom we selected on the basis of their involvement in administering or implementing the CTG. By using a qualitative sampling method called snowball sampling (14), we first interviewed administrators and leaders and then asked these participants to identify additional people involved in CTG implementation.

All key informants we contacted agreed to participate (Table 1). Throughout this process we ensured equitable representation from the 5 regions. At least 2 members of our research team conducted all interviews (L.P.W., C.M., C.L.A., T.V., P.A.H.), either in person (n = 3) or by telephone (n = 31), using individually tailored discussion guides designed to gather feedback about various aspects of CTG objectives and implementation. We used document review and evaluation tools developed by Cheadle et al (15) to inform our general discussion guide, and we verified the guide with WA DOH leadership before use. We emailed discussion guides to each participant in advance, and discussed the goals of the process evaluation with participants before starting each interview. Interviews lasted 45 to 60 minutes, were recorded with permission, and professionally transcribed (Proof Positive Transcriptions, Garland, Texas); all participants gave permission to be recorded. We regularly discussed data saturation, and we continued interviewing until we reached saturation.

**Table 1:** Study Participants (N = 34), Community Transformation Grant Process Evaluation, 2014

Affiliation	No. (%)
**Washington State Department of Health**	14 (41)
**Regional local health jurisdictions**	11 (32)
**External consultants**	4 (12)
**Regional partners**	5 (15)
**Sex**	
Male	13 (38)
Female	21 (62)

### Analysis

We used directed content analysis (16) to guide our data analysis methodology. Directed content analysis is a process by which researchers use existing theories or prior research to develop the initial coding scheme (16). Following each interview we wrote brief case summaries; we developed our initial coding structure based on the important constructs in these summaries. We revised and finalized our coding structure after coding the transcripts of the first and second interviews. We used qualitative data analysis software (Atlas.ti Scientific Software Development GmbH) to code each interview. To ensure accuracy and consistency, 1 member of the research team coded every interview (L.P.W.); 1 of 2 secondary coders (C.M., C.L.A.) also independently coded and reconciled 19 interviews (56%). Researchers reviewed coded data, identified themes, and discussed and agreed on findings. We gave all participants the opportunity to provide feedback on a draft of our findings.

## Results

Participants said that implementing policy and systems change work through regional collaboration was complex. We describe the primary factors that participants identified as challenging and facilitating in CTG implementation. These factors were identified by most participants across organizations and roles. We have grouped them based on whether participants identified them as occurring in the planning and building stages of the regional model or during implementation of the regional work. Exemplar quotes from participant interviews are in Table 2.

**Table 2:** Participant Quotes on Regional Implementation of the Community Transformation Grant in the 5 Regions, Washington State, 2014

Factor	Quotes
**Planning and building a regional model**	
**Challenges**	
Buy-in	“When the Community Transformation Grant was implemented, there wasn’t a specific broad-level engagement with the local health jurisdictions about the idea of a regional [model].”
Geography	“The geographic regions don’t lend themselves to be a Hub.”
	“It became prohibitive to get everybody together in a room.”
Governance and administrative structure	“One thing that I don’t know we’re ever going to overcome . . . is expecting a Hub to subcontract with their other peers.”
**Facilitators**	
Shared history and infrastructure	“Whatever regional model you have . . . it really needs to fit the working relationships of that area.”
Leadership and relationships	“It takes the right leadership to make this work.”
	“A lot of it depends on kind of what relationships and connections were existing beforehand.”
Shared vision and common goals	“All of the local health jurisdictions really are interested in healthy communities work. . . . That has really helped collaboration.”
**Implementing a regional model**	
**Challenges**	
Communication	“In the interest of time and efficiency and clarity, it’s hard to ask clear questions and receive clear answers when there is a go-between.”
Funding to support administrative and staffing costs	“We have had to put our own resources into this.”
	“All of the staff had full plates. The Community Transformation Grant really just bought an arm of someone. There is no way that we could hire a full body.”
**Facilitators**	
Availability of additional funds	“Having the opportunity to leverage some of the other funds that weren’t included in the Community Transformation Grant has been a real benefit. . . . It’s definitely a way to bring in those other counties.”
Direct technical assistance and training	“We would not be where we were if it weren’t for [the external consultants].”

### Planning and building a regional model

#### Challenges

**Buy-in from local leaders and regional partners.** The limited timeline given for WA DOH to develop its CTG funding application precluded broad consultation with local partners. Participants uniformly said that implementation could have been more effective with greater local-level engagement during the initial planning process. Participants identified the opportunity to provide input in the initial planning phases as a way to increase local buy-in. Participants from LHJs specifically identified the following areas where they would have liked to provide greater input: regional geographic boundaries, fiscal management strategies, determination of specific evidence-based interventions for implementation, and strategies for ensuring equitable regional authority.

**Regional geography.** Large geographic regions posed a substantial challenge to regional collaboration. Difficulty meeting in person because of distance or topography impeded collaboration across county borders. Larger regions were also less likely to benefit from strong pre-existing relationships and struggled to coalesce around shared interests and concerns. Participants felt that smaller, more topographically cohesive geographic regions could have increased their ability to implement work plans.

**Governance and administrative structure.** The governance structure and policies within each regional lead LHJ played a substantial role in its ability to manage contracting and fiscal responsibilities. The willingness of county boards of health within each region to collaborate influenced the timeliness and effectiveness of implementation. Participants expressed the importance of understanding the political and administrative structure of regional leads before distributing funds.

#### Facilitators

**Shared regional history and infrastructure.** Regions that were the most successful in collaborating had a history of doing so and were more likely to have shared infrastructure already. Participants identified partnerships, such as Educational School Districts or emergency preparedness work, and shared infrastructure, such as transportation and hospital systems, as pre-existing regional partnerships that supported collaboration on chronic disease prevention. Previous collaboration between LHJs provided natural partnerships that did not require the same time or effort to nurture and develop compared with new partnerships.

**Strong local leadership and collaborative relationships.** Strong regional leadership and staff were vital to engaging partners necessary for accomplishing policy and systems change; the leaderships’ ability to convene regional partners and communicate effectively with different sectors facilitated work plan implementation and generated regional momentum. Furthermore, positive personal and professional relationships between staff and regional partners facilitated collaboration and communication within regions as well as between regional coordinators and WA DOH. Existing relationships increased the speed at which regions could implement policy and systems change work and convene regional partners.

**Shared vision and common goals.** A shared region-wide vision and common goals compelled regional partners to act more quickly and to efficiently develop and coalesce around work plans. Participants felt that building regions around shared vision and goals and supporting regions to identify commonalities are essential to facilitating success.

### Implementing a regional model

#### Challenges

**Communication.** An important issue for collaborative regional models is overcoming challenges related to communication by learning to communicate effectively through new or different channels. Many participants felt that new regional communication channels were unclear and impeded their ability to efficiently implement work. Without open and frequent communication, participants reported that they relied on external personal relationships to receive information and advice; this fostered distrust among partners and staff as well as disparate levels of support across regions. Participants expressed the desire for greater communication throughout the implementation process. Participants also felt better communication could have fostered increased access for regions to external technical assistance and increased support from WA DOH staff.

**Funding to support administrative and program staff.** Serving as a CTG grant administrator proved costly and time-consuming for the regional leads. Several regional leads felt that the administrative stipend provided through CTG was insufficient to cover actual costs. Limited resources to support adequate staffing negatively affected regions’ abilities to implement policy and systems changes. This was a particular challenge in rural areas, where it was especially difficult to attract and retain trained staff.

**Work plan flexibility.** Participants reported that when working collaboratively across regions, there was a substantial need for work plan flexibility. Participants found that the structure of the grant funding led to difficulty changing course when a particular intervention proved unfeasible. Participants felt that building work plan flexibility into the initial grant structure could enable regions to develop and implement policies and programs that best fit their communities.

#### Facilitators

**Availability of additional funds to leverage regional participation.** Leveraging additional funding for peer counties not targeted through CTG was an important means of encouraging regional collaboration. Regional leads who could offer funds to the nontarget counties in their region experienced greater engagement. Participants also felt that additional, sustainable funding was an important resource for attracting nontraditional partners (eg, the private sector).

**Direct technical assistance and training.** Technical assistance provided by independent external consultants was available to help regions implement program-related evidence-based practices, conceptualize policy and systems change work in their region, and build regional and cross-jurisdictional collaboration. Participants in regions that received a high level of technical assistance felt it provided valuable expertise and additional capacity to advance policy and systems changes. Furthermore, several participants felt that direct technical assistance could play a key role in supporting regions to develop sustainability plans and secure long-term funding.

## Discussion

In this process evaluation we identified specific challenges and facilitators to building and implementing a regional public health model to reduce chronic disease risk factors through policy, environment, and systems changes. Lessons learned in Washington State may help other states plan, build, and implement similar regional models.

Many of the challenges and facilitators we identified are similar to those described in literature on cross-jurisdictional emergency preparedness planning. However, achieving policy and systems changes differs from other cross-jurisdictional efforts given its long-term nature and relative newness to public health (17–19). Common challenges to regional work include issues surrounding regional authority and local partisan politics; it is difficult for decentralized public health systems to restructure work models when local leaders and authorities are not supportive (2). Early buy-in and ongoing involvement from local political and public health leaders is essential for effective implementation of regional public health work. Common facilitators to regional work include a strong history of personal and professional relationships, strong leadership, shared infrastructure, common vision and goals, open communication, clear memorandums of understanding or written agreements between regional partners, and the ability to leverage additional funding to support participation (2,5).

Policy and systems change work can be contentious. Participants found engaging stakeholders and obtaining buy-in from local leaders and regional partners to be an arduous process. This underscores the importance of developing regional models with the intention to maintain these relationships once established. Participants in this evaluation said that policy and systems changes require time, ongoing and sustainable funding, and the flexibility to change course if needed.

Effective implementation of regional models requires that funders and administrators engage local partners early and often. Before determining geographic regions and regional work plans, it is important that funders and administrators consider historical relationships, political tensions within and across regions, and objections to regional work. Obtaining buy-in and assessing local environments is time-intensive. Funders can support the process by providing sufficient time for local engagement during the grant proposal–writing process, at the start of the funding period, or both.

Stable, ongoing, and flexible funding is critical to support regionalized policy and systems change work (4,9). Effective regional public health efforts require that LHJs cultivate nontraditional relationships. Committed, ongoing funding helps build credibility and supports sustainable partnerships by allowing LHJs to commit to long-term regional partnerships. Administrators should support regions to develop sustainability plans that will enable regional work to continue after grant funding has ended. Given the political nature of policy and systems change work, it is important that funders and administrators allow flexibility throughout the implementation of regional models so that regions can adapt and change course if selected strategies prove unfeasible in their communities.

As a process evaluation, we did not evaluate CTG outcomes across regions or the health effects of the policy and systems changes implemented in Washington State through CTG. Rather, this research is limited to the process of implementation and the factors that affected regions’ abilities to implement the work. Although our findings will not provide midterm feedback for WA DOH because of the early termination of CTG funding, they remain meaningful given Washington’s intent, and the incentives, to continue regional efforts.

These results are not uniformly generalizable. We need future research and ongoing evaluation to develop an evidence base for the efficacy of regional public health models as a strategy to prevent and reduce chronic disease. We also need further research to understand the financial implications and potential cost savings of resource-sharing across local public health jurisdictions.
